# An *Eimeria maxima* Antigen: Its Functions on Stimulating Th1 Cytokines and Protective Efficacy Against Coccidiosis

**DOI:** 10.3389/fimmu.2022.872015

**Published:** 2022-05-20

**Authors:** Chen Chen, Yue Zhang, Jianhua Liu, Mingyue Wang, Mingmin Lu, Lixin Xu, Ruofeng Yan, Xiangrui Li, Xiaokai Song

**Affiliations:** Ministry of Education (MOE) Joint International Research Laboratory of Animal Health and Food Safety, College of Veterinary Medicine, Nanjing Agricultural University, Nanjing, China

**Keywords:** *Eimeria maxima*, Th1 cytokines stimulating antigen, EmARM-β, protective efficacy, DNA vaccine

## Abstract

A consensus is that the Th1 immune response plays a predominant role against avian coccidiosis. Therefore, an antigen with the ability to induce Th1 cytokine responses is an ideal candidate for the development of coccidiosis vaccines. In our previous study, EmARM-β, a Th1 cytokines-stimulating antigen, was screened from the cDNA expression library of *Eimeria maxima* (*E. maxima*). Herein, we verified its stimulative effects on Th1 cytokine productions and evaluated its protective efficacy against *E. maxima* infection. Recombinant EmARM-β protein was expressed, and eukaryotic expression plasmid pVAX1-EmARM-β was also constructed for the immunization of birds. An immunofluorescence assay was performed to detect the native form of EmARM-β protein in the stage of sporozoites. Expressions of specific transcription factors and cytokines in immunized chickens were measured using qPCR and ELISA to verify its stimulating function on Th1 cytokines. Specific IgG antibody levels and T lymphocyte subpopulation in the immunized chickens were detected using ELISA and indirect flow cytometry to determine induced immune responses. The results showed that EmARM-β native protein is massively expressed in the sporozoites stage of *E. maxima*. Effective stimulation from the EmARM-β antigen to T-bet and Th1 cytokines (IL-2 and IFN-γ) was observed *in vivo*. After being immunized with rEmARM-β or pVAX1-EmARM-β, significant promotion to the proportion of CD4^+^ and CD8^+^ T cells and the level of antigen-specific IgG antibodies in immunized chickens was also observed. Furthermore, vaccination with rEmARM-β antigen or pVAX1-EmARM-β resulted in alleviated weight loss and enteric lesion, reduced oocyst output, and higher anticoccidial index (ACI) in challenged birds. These results indicate that EmARM-β antigen can effectively stimulate the expression of Th1 cytokines and initiate host immune responses, providing moderate protective efficacy against *E. maxima*. Notably, EmARM-β protein is a promising candidate for developing a novel anticoccidial vaccine.

## Introduction

As a parasitic enteric disease, avian coccidiosis can cause malabsorption, poor feed-conversion performance, and body weight loss to worldwide chickens. The global economic loss of chicken coccidiosis has been estimated to be £10.4 billion in 2016 (1). Currently, the primary control strategies against *Eimeria* parasites mainly rely on feeding anticoccidial drugs or live parasite vaccination. However, with the rising public attention in drug residues and the rapid generation of drug-resistant strains of *Eimeria* species, mixing anticoccidial drugs with feed has been banned gradually worldwide (2). Moreover, owing to its inherent limitations, including production limitation, cost issues, and potential pathogenicity, the development of live vaccines is full of frustrations (3). Recently, novel anticoccidial strategies, including recombinant subunit vaccines, have arisen great interest. The core of developing a novel vaccine is identifying the antigens which can induce an effective immune response against coccidiosis. However, multiple developmental stages of *Eimeria* spp. and the antigenic variation and modification during specific developmental stages make it challenging to obtain precious candidate antigens (4, 5).

T helper (Th) cells are considered key mediators of host immune response. They can be divided into different Th lineages according to their function and unique cytokine production profiles, such as Th1, Th2, Th17, and inducible T regulatory (iTreg) cells (6). Th1 cytokines, such as IL-2 and IFN-γ, have been confirmed to be essential in resisting avian coccidiosis. For example, Lillehoj et al. found that the the sporozoites development of *Eimeria tenella in vitro* was inhibited by chicken cells expressing recombinant chicken IFN-γ (7). What’s more, oocyst production and weight loss caused by infection of *Eimeria acervulina* in chickens treated with recombinant chicken IFN-γ were significantly improved. Another research conducted by this group confirmed that the IFN-γ gene injected into chicken fibroblasts can significantly inhibit the growth of subsequently infected sporozoites (8). Recently, a transgenic *Eimeria mitis* line expressing chicken IL-2 was constructed by Li et al. (9). They found that immunization with this transgenic line can induce elevated cellular immune responses and reduce oocyst output in birds. A similar adjuvant function was also found in IFN-γ. The insertion of this cytokine into the DNA vaccine exhibits an excellent immune effect enhancement (10, 59, 60). These results showed the critical roles that Th1 cytokines play against the infection of *Eimeria* species. Therefore, an antigen that possesses a stimulating effect on Th1 cytokines may be a valuable candidate for developing a novel anticoccidial vaccine.

In our preliminary work, EmARM-β, a Th1 cytokine stimulating antigen, was obtained from cDNA library of *E. maxima* through expression library immunization (unpublished data). In this study, we further verified the stimulating effect of EmARM-β on Th1 cytokines *in vivo* and evaluated its protective efficacy against *E. maxima* infection through challenge trials.

## Materials And Methods

### Animals, Parasites, and Reagents

Clean and strictly sterilized cages and coccidia-free rooms were employed for the rearing of female Hy-Line chickens (1 day old). All chickens have free access to food and water without coccidiostats until the end of the experiment. Eukaryotic expression vector pVAX1 was purchased from Invitrogen (Carlsbad, California). Prokaryotic expression vector pET-28a, uninfected chicken serum, and chicken antiserum against *E. maxima* were preserved in our lab (10, 11). Sporulated oocysts of *E. maxima* were stored in 2.5% potassium dichromate at 4°C. The propagation of sporulated oocysts was conducted in chickens seven days before the challenge trial. Female SD rats were purchased from Qinglongshan animal breeding farm, Nanjing, China. The Committee on Experimental Animal Welfare and Ethics of Nanjing Agricultural University reviewed and approved all animal studies and protocols.

### Gene Amplification and Expression Plasmid Construction of EmARM-β

Total plasmids were extracted from the EmARM-β single clone library. According to the complete open reading frame (ORF) on NCBI, the software “Primer Premier 5” (Premier, Canada) was used for the design of restriction enzyme-anchored (underlined) primers: *Bam*HI anchored forward primer, 5’-CGCGGATCCATGCATCCGTGGGCTGC-3’; *Hin*dIII anchored reverse primer, 5’-CCCAAGCTTTCAGTCTGCCAGACGAACCAA-3’. The polymerase chain reaction procedure was carried out as follows: initial denaturation at 94°C for 5 min; followed by denaturation at 94°C for 30 s, annealing at 58-62°C for 30 s and extension at 72°C for 50 s, total 35 cycles; finally plus an extension at 72°C for 7 min. After electrophoresis with an agarose gel, the produced EmARM-β gene was recycled utilizing Gel Extraction Kit D2500 (OMEGA, Norcross, Georgia). The recovered products were ligated into pET-28a prokaryotic expression vector digested with *Bam*HI and *Hin*dIII to generate recombinant plasmid pET-28a-EmARM-β. Then recombinant plasmid was transformed into *E. coli* BL21 competent cells. Verification of recombinant products was conducted by endonuclease digestion and sequence analysis with BLAST (http://www.ncbi.nlm. nih.gov/BLAST./).

To construct eukaryotic expression plasmid pVAX1-EmARM-β, the primers of EmARM-β were designed by the software “Primer Premier 5” (Premier, Canada) according to the sequence published on NCBI (XM_013482460.1). Restriction enzyme sites were underlined: *Bam*HI anchored forward primer, 5’-CGCGGATCCATGCATCCGTGGGCT-3’; *Xho*I anchored reverse primer, 5’-CCGCTCGAGTCAGTCTGCCAGACGAAC-3’. PCR amplification and product recycling were carried out as stated above. Obtained PCR fragments were ligated into eukaryotic expression plasmid pVAX1 digested with *Bam*HI and *Xho*I. Endonuclease digestion and sequence analysis were performed to determine the recombinant products. Recombinant plasmid pVAX1-EmARM-β and empty vector pVAX1 for the vaccination trials were prepared using FastPure EndoFree Plasmid Maxi Kits (Vazyme, Nan-jing, China) according to the manufacturer’s instructions.

### Purification and Western Blot Analysis of EmARM-β Recombinant Protein

rEmARM-β was expressed through pET-28a-EmARM-β transformed *E. coli* BL21 cells by inducing with isopropyl-D-thiogalactopyranoside (500mM). According to the manufacturer’s instructions, the recombinant protein was purified with the Ni2+-nitrilotriacetic acid (Ni-NTA) column (GE Healthcare, USA). Endotoxin of purified rEmARM-β was removed by ToxinEraser™ Endotoxin Removal Kit (GenScript, China). Then an SDS-PAGE assay was conducted to determine expressed recombinant EmARM-β protein.

An immunoblot assay was carried out with chicken antiserum against *E. maxima.* In brief, the purified recombinant protein was transferred to the nitrocellulose (NC) membrane after the SDS-PAGE assay.

After washing three times with phosphate buffer saline with 0.05% Tween-20 (PBST), the NC membrane was blocked with 5% bovine serum albumin (BSA) at room temperature (RT) for 1.5 h. Then the NC membrane was incubated with chicken antiserum against *E. maxima* (1:100 dilution) or His-tag Mouse Monoclonal antibody (1:8000 dilution; Proteintech, Wuhan, China) at 37°C for 3 h. Meanwhile, naïve chicken serum was used as the negative control. Next, NC membranes were washed three times and then incubated with horseradish peroxidase (HRP)-conjugated goat anti-chick IgG (1:3000 dilution) or HRP-conjugated anti-mouse (1:10000 dilution, Thermo Fisher Scientific, Waltham, MA, USA) at RT for 2 h. After washing with PBST, color rendering was performed utilizing an ECL chemiluminescence detection kit (Thermo Scientific, Waltham, MA) to detect the bound antibody.

SD rats (one-month-old) were subcutaneously injected with 300 μg rEmARM-β to generate antiserum for rEmARM-β. At primary immunization, rEmARM-β was emulsified with an equal volume of Freund’s Adjuvant Complete (Sigma-Aldrich, Merck KGaA, Darmstadt, Germany). Then, four more injections were performed with rEmARM-β emulsified with Freund’s Adjuvant Incomplete (Sigma-Aldrich) with intervals of 7 days. Blood was collected from the fundus venous plexus one week after the fifth vaccination. Indirect ELISA was carried out to determine the titers of antiserum. Naïve rat serum was collected and used as the negative control.

### Expression of EmARM-β in Sporozoite Stage of *E. maxima* by Immunofluorescence

*E. maxima* sporozoites were smeared on poly-L-lysine treated glass slide and stayed the slide at 4°C overnight. Then the sporozoites were fixed on the slides using 4% paraformaldehyde in TBS (Tris-HCl buffer saline) for 10 min at RT. The slides were washed three times with PBST, 6 min each time. Pre-dissolved 1% TritonX-100 was used for permeabilization at RT for 10 min. Subsequently, slides were washed three times with PBST and blocked with 5% BSA in PBST at 37°C for 1-2 h. After washing with PBST, the rat antiserum against rEmARM-β (1:200 dilution) was used to incubate with the slides at 37°C for 2 h. Slides treated with serum from non-immunized rats served as negative controls. After three washes with PBST, Cy3 conjugated goat anti-rat IgG antibody (dilutions 1:1000) was incorporated into the slides in darkness for 40 min. After three washes in PBST, DAPI (4′, 6-diamidino-2-phenylindole, 10 μg/ml in PBS) (Beyotime, Shanghai, China) was added on slides for staining. Slides were examined by fluorescent microscopy (Olympus, Japan).

### Stimulation Effects of EmARM-β Antigen on Th1 Cytokines *In Vivo*

Fourteen-day-old chickens were randomly assigned into five groups, with 10 chickens per group. Chickens from experimental groups were intramuscularly injected with 100 μg of recombinant protein or recombinant plasmid of EmARM-β, respectively. Control groups were injected with 100 μg of pET-28a tag protein, 100 μg of pVAX1 plasmid, or PBS, respectively. Six days later, blood samples were collected by cardiac puncture to obtain chicken serum and peripheral blood mononuclear cell (PBMC).

The levels of transcription factors T-bet and GATA3 were detected using qPCR assays. GAPDH gene was served as the internal control (12). According to the sequence published on NCBI (T-bet: CB016768; GATA3: NM.0010084441.1), specific primers were designed as listed in **Table 1**. Total RNA was extracted utilizing Total RNA Kit II (OMEGA) from chicken PBMC prepared before. Then cDNA was produced by RT-PCR and used as templates for qPCR amplification. The reaction system contains 2 μL of template cDNA, 0.4 μL of forward and reverse primers, 10 μL of 2×ChamQ SYBR qPCR Master Mix and 7.2 μL of sterile water. Meanwhile, sterile water was used as the negative control. Triplicated samples were set in each assay. We employed 2^-ΔΔCT^method to estimate the relative quantification of transcription factor compared with the internal control (n-fold change to the water control group) (13). Serially diluted cDNA was used to validate amplification efficiencies of the primers for transcription factors and reference gene (14).

**Table 1:** Sequence of the primers used in real-time quantitative RT-PCR.

RNA target	Primer sequence	Accession NO.	Amplification efficiency (%)	Correlation coefficients (r^2^)
GAPDH	GGTGGTGCTAAGCGTGTTAT	K01458	100.74%	0.9917
	ACCTCTGTCATCTCTCCACA			
IL-2	TAACTGGGACACTGCCATGA	AF000631	102.44%	0.9921
	GATGATAGAGATGCTCCATAAGCTG			
IL-4	ACCCAGGGCATCCAGAAG	AJ621735	99.09%	0.9936
	CAGTGCCGGCAAGAAGTT			
IL-10	GGAGCTGAGGGTGAAGTTTGA	AJ621614	99.19%	0.9923
	GAAGCGCAGCATCTCTGACA			
	GAGAACTGCCTTGCCTAACA			
IFN-γ	AGCTGACGGTGGACCTATTATT	Y07922	103.07%	0.9868
	GGCTTTGCGCTGGATTC			
TGF-β4	CGGGACGGATGAGAAGAAC	M31160	102.79%	0.9815
	CGGCCCACGTAGTAAATGAT			
T-bet	CCGAGACACAGTTCATTGCT	CB016768	101.61%	0.9923
	AGTTATCCCGGAAGCCTTTG			
GATA3	GGCTGGACGGGAGCAAAG AGCAGGCGGGTAAACGGA	NM.0010084441.1	99.25%	0.998

Chicken serum was prepared from blood samples collected six days after vaccination. Enzyme-linked immunosorbent assay (ELISA) was conducted to measure the expression of associated cytokines according to the previous study (15). Briefly, we diluted rEmARM-β to 10 μg/mL with 50mM carbonate buffer. Then 100 μL of dilutions were coated in each well of flat-bottomed 96-well plates at 4°C overnight. After washing the plate five times with PBST, 100 μL of 5% BSA was added to the plate for blocking. Subsequently, a 100 μL mixture of serum samples and PBS (1:50 dilution) was incubated at 37°C for 1.5 h. Serum from non-immunized chickens was served as the negative control. After five washes, 100 μL of HRP-conjugated goat anti-chicken IgG antibody (dilutions 1:3000) was added to each well of the plate. Then the plate was incubated at 37°C for 2 h. An incubation in darkness was performed at RT for 15 min after adding 100 μL 3,-3’,5,-5’-tetramethylbenzidine (TMB). Then color development was determined at OD450 with a spectrophotometric.

### The Cellular and Humoral Immune Responses Induced by EmARM-β in Chickens

Fourteen-day-old chickens were randomly divided into four groups, with 10 chickens per group. Chickens from two experimental groups were intramuscularly injected with 200 μg of rEmARM-β or 100 μg of recombinant plasmid pVAX1-EmARM-β, respectively. Two control groups were respectively injected with 100 μg of pVAX1 plasmid and 200 μL of PBS, similar to experimental chickens. One more vaccination was performed at 7 days after the primary immunization. Blood samples and spleen lymphocytes were collected from five chickens which were randomly chosen from each group at the age of 21 days and 28 days old.

Spleen lymphocyte was collected from five chickens of each group seven days post each vaccination as previously described (16). In brief, spleens were cut into scraps with sterile scissors and were ground thoroughly. According to the manufacturer’s protocol, the separation solution (TBD, Tianjin, China) was used to prepare the splenocytes. The concentration of spleen cell suspensions was adjusted to 1×10^7^ -1×10^8^ cell/mL. Cell suspensions were divided into 5 tubes, with each tube containing 1×10^7^ splenocytes in 100 μL PBS buffer. Among these five tubes, two tubes were chosen as controls. One of them was stained with 2 μL of anti-chicken CD3 mouse antibody. The other was stained with 2 μL of anti-chicken CD4 or CD8 mouse antibody. Another two samples were dually stained with 2 μL of anti-chicken CD3 antibody and 2 μL of anti-chicken CD4/CD8 antibody. The last tube was non-staining. All tubes were incubated in darkness at 4°C for 45 min. Then T cell subpopulation analysis was conducted by a FACS Calibur flow cytometer (BD Biosciences, Franklin Lakes, NJ).

Indirect ELISA was performed to measure the serum IgG antibody level following the method described in the *in vivo* experiment. Seven days after each immunization, chicken serum was prepared from blood samples collected by cardiac puncture. Recombinant protein pET-28a-EmARM-β was diluted to 20 μg/mL and coated in flat-bottomed 96-well plates. Previously prepared chicken serum (dilutions 1:100) and HRP-conjugated goat anti-chicken IgG antibody (1:3500 dilution) were used separately as the primary and secondary antibodies in ELISA. Serum from non-infected chickens served as negative controls.

### Protective Efficacy Assessment of EmARM-β Against *E. maxima*

Two immunization-challenge trials were conducted to assess the protective efficacy of rEmARM-β (Trial 1) and pVAX1-EmARM-β (Trial 2). In Trial 1, 60 fourteen-day-old chickens were randomly and equally divided into unchallenged control, challenged control, and experimental groups. Unchallenged control birds received no treatment. Birds in the challenged control group were challenged with oocysts without being vaccinated with protein. Chickens of the experimental group were intramuscularly immunized with 200 μg of rEmARM-β. In trial 2, 80 fourteen-day-old chickens were randomly divided into 4 groups (20 chickens per group), i.e., unchallenged control, challenged control, pVAX1 control, and experimental groups. The unchallenged control and challenged control groups were treated the same way in Trial 1. The experimental and pVAX1 control groups were intramuscularly vaccinated with 100 μg of pVAX1-EmARM-β plasmid and 100 μg of pVAX1 plasmid, respectively.

A booster with the same dose was given one week after the primary immunization. Chickens at 28 days old were orally challenged with 1×10^5^ oocysts of *E. maxima*. Chickens were humanely sacrificed for data generation six days later, including enteric lesion score, oocysts shedding, body weight gain, and anticoccidial index (ACI). Body weight gain was the chicken weight difference between challenge and execution. Oocyst counting was carried out using a McMaster chamber following the previous method (17). According to the lesion scoring techniques reported by Johnson and Reid (18), a numerical scale from normal to severe (0 to 4) was used for scoring the enteric lesions (the midgut area located above and below the yolk sac diverticulum) (18). Oocysts shedding decrease ratio was measured as the following formula: (mean oocysts amount of the challenged control group - that of the vaccinated groups)/oocysts amount of control group×100%. ACI was calculated according to the previous method (19).

### Statistical Analysis

Independent-samples T-test and one-way ANOVA Duncan test were adopted to analyze statistic differences between groups with SPSS statistical package (SPSS for Windows 13, SPSS Inc., Chicago, IL, USA). Differences between groups at *p* < 0.05 were considered significant.

## Results

### Gene Amplification and Protein Purification of EmARM-β

**Figure 1:**
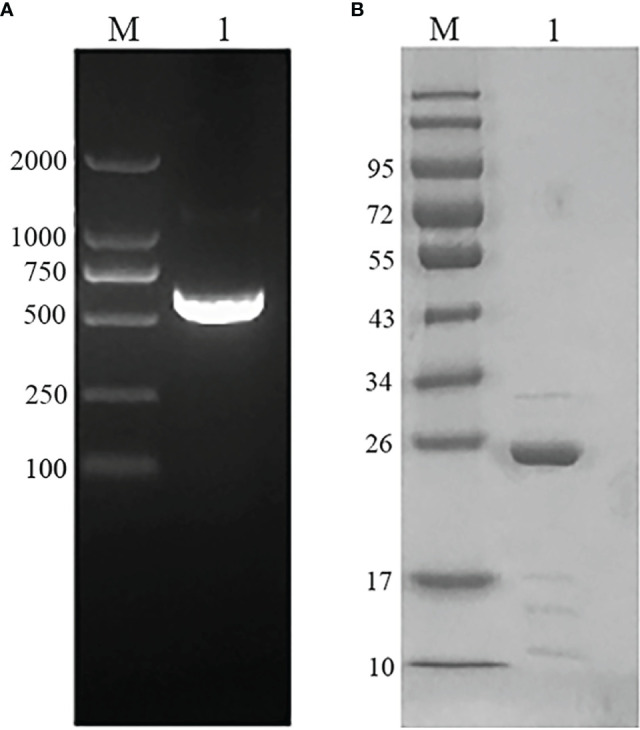
Gene amplification and protein purification of EmARM-β. **(A)** Amplification of EmARM-β gene. M: DNA marker DL2000. Lane 1: the amplified EmARM-β gene from cDNA library. **(B)** Purification of recombinant protein EmARM-β detected by SDS-PAGE. M: protein mid-molecular weight marker. Lane 1: Purification of recombinant protein pET-28a-EmARM-β.

The plasmid was extracted from the EmARM-β single clone library and served as templates for PCR amplification. The resulting PCR clones were ligated into prokaryotic expression vector pET-28a. The amplified gene (612bp) (**Figure 1A**) shared a 100% nucleotide homology with the sequence in GenBank (Sequence ID: XM_013482460.1). After transferring into *E. coli* BL21, recombinant plasmid pET-28a-EmARM-β was induced for expression. The purification of rEmARM-β was performed using the Ni-NTA column, and the protein purification results were shown in **Figure 1B** on an SDS-PAGE gel. A single band with the size of approximately 26kDa was observed, suggesting rEmARM-β was well purified.

### Detection of Recombinant and Native EmARM-β by Western Blot and Immunofluorescence Assay

**Figure 2:**
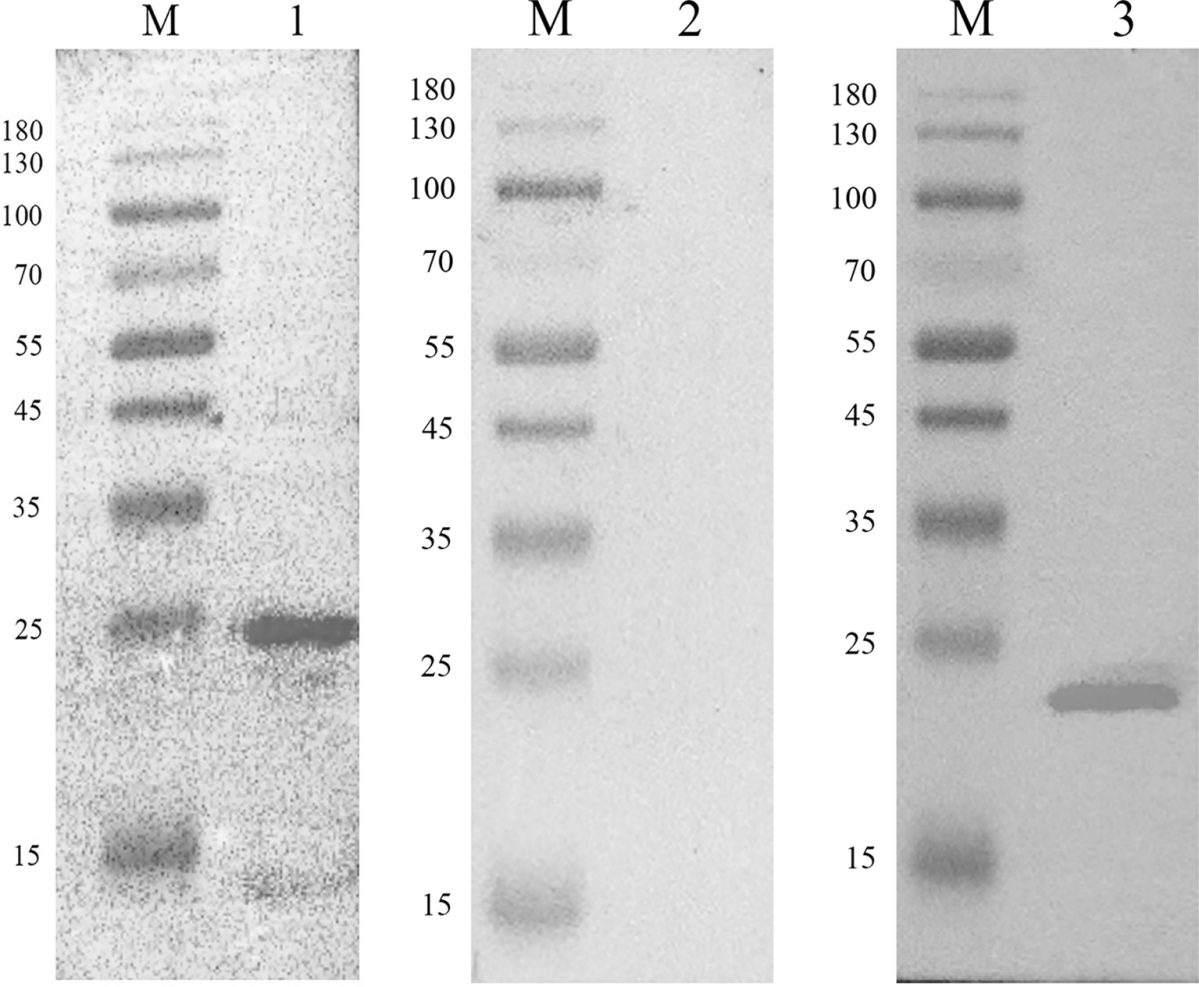
Immunoblot analysis of recombinant protein pET-28a-EmARM-β Western blot analysis of recombinant protein pET-28a-EmARM-β. M: protein mid-molecular weight marker. Lane 1: The recognition of recombinant protein pET-28a-EmARM-β by the chicken serum against *E. maxima*. Lane 2: The recognition of recombinant protein pET-28a-EmARM-β by negative chicken serum. Lane 3: The recognition of His-Tag in recombinant protein pET-28a-EmARM-β by His-tag Mouse Monoclonal antibody.

The recombinant EmARM-β protein was identified through Western blot assays using *E. maxima* antiserum and His-tag mouse monoclonal antibody separately. As shown in **Figure 2**, *E. maxima* antiserum recognized a single band with the molecular weight of approximately 26 kDa (**Figure 2**, Lane 1), which was consistent with the molecular weight of rEmARM-β. Meanwhile, the His-tag mouse monoclonal antibody recognized a single band whose size was slightly smaller than 26 kDa (**Figure 2**, Lane 3). In contrast, the serum from control birds (negative serum) did not recognize rEmARM-β (**Figure 2**, Lane 2).

**Figure 3:**
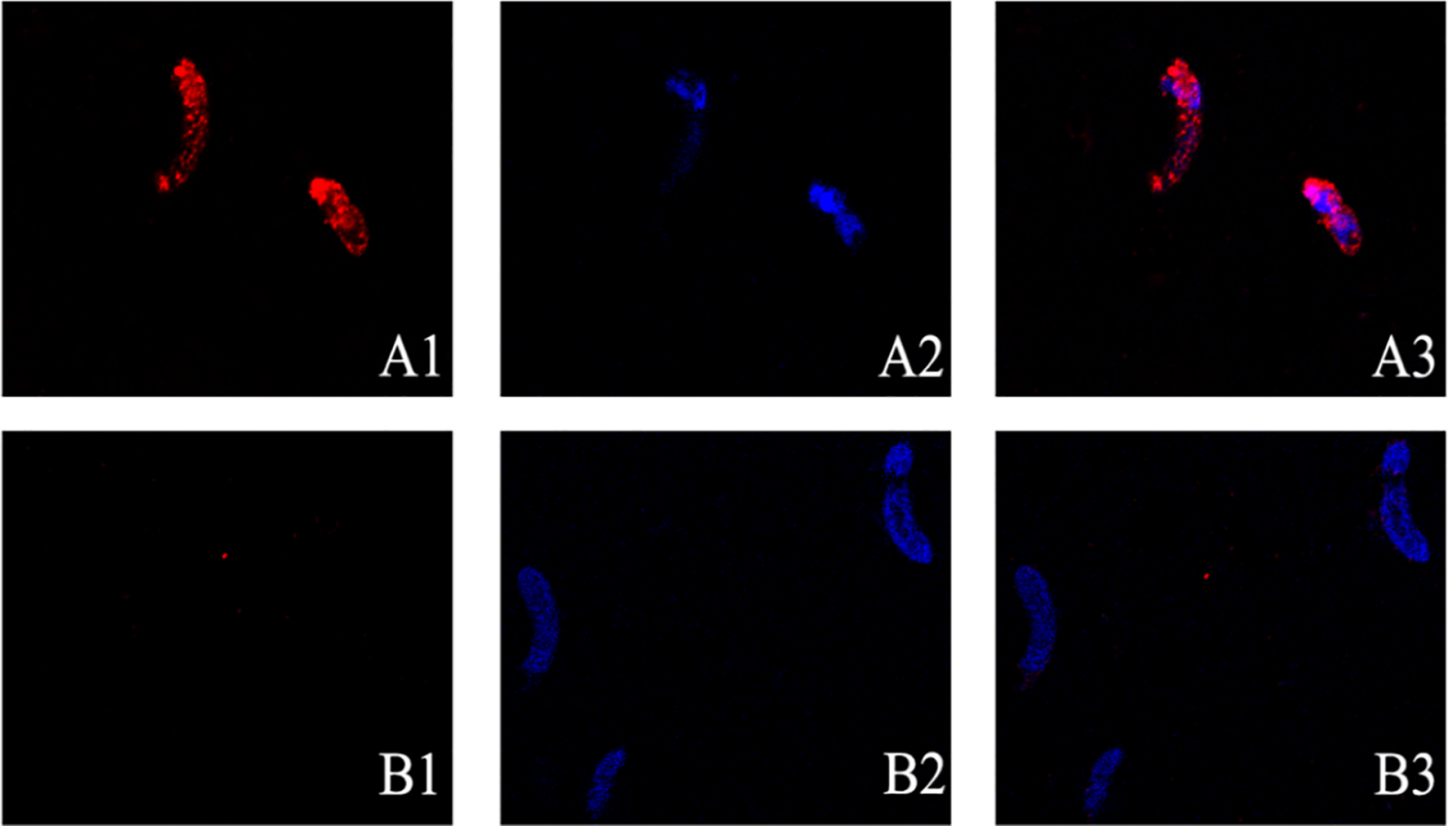
Expression of EmARM-β in sporozoites stage of *E. maxima*. **(A)** The sporozoites were detected by anti-rEmARM-β rat serum. A1: Sporozoites were dyed by Cy3; A2: The nuclei were detected by DAPI; A3: Merge. **(B)** The sporozoites were detected by negative rat antibodies. B1: Sporozoites were dyed by Cy3; B2: The nuclei were probed by DAPI; B3: Merge.

Expression of EmARM-β in the sporozoite stage was detected by immunofluorescence assay (**Figure 3**). Antiserum from immunized rats was used to detect native EmARM-β protein in sporozoites. Prominent red fluorescence was observed in the sporozoite of *E. maxima* incubated with anti-rEmARM-β rat serum (**Figure 3**, A3). No red fluorescence was observed in sporozoites from negative control (**Figure 3B**). The results from immunofluorescence assays indicated an intensive expression of EmARM-β in the sporozoites stage of *E. maxima* (**Figure 3**, A3).

### Stimulation Effect of EmARM-β Antigen on Th1 Cytokines *In Vivo*

**Figure 4:**
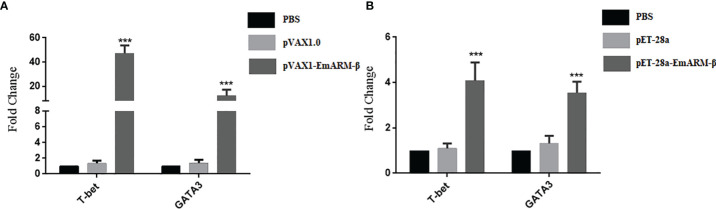
Level changes of T-bet and GATA3 in chickens induced by EmARM-β. Specific transcription factor levels measured by quantitative PCR. **(A)** Level measurement of specific transcription gene induced by recombinant plasmid pVAX-1-EmARM-β. **(B)** Level measurement of specific transcription gene induced by recombinant protein pET-28a-EmARM-β. ***p < 0.001.

**Figure 5:**
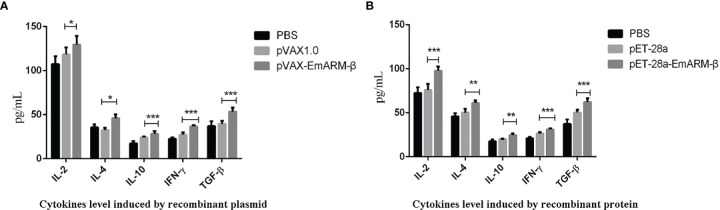
Concentration measurement of serum cytokines induced by EmARM-β. Serum cytokines concentration induced by EmARM-β was measured by ELISA assay. **(A)** Cytokines level induced by recombinant plasmid pVAX1-EmARM-β. **(B)** Cytokines level induced by recombinant protein pET-28a-EmARM-β. *p < 0.05; **p < 0.01; ***p < 0.001.

Six days after immunization, blood samples collection was conducted to measure the levels of specific transcription factors and related cytokines by qPCR and ELISA, respectively. As shown in **Figure 4**, the levels of T-bet and GATA3 in chickens immunized with pVAX1-EmARM-β plasmid and rEmARM-β were significantly higher than those of control groups (*p* < 0.05). No significant difference was observed between PBS control and pVAX1 or pET-28a control (*p >* 0.05). In the measurement of sera cytokines concentration, EmARM-β exhibited a significant stimulation to Th1 cytokines (IFN-γ and IL-2) compared with control groups (*p* < 0.05) (**Figure 5**). Besides, the levels of Th2 and Treg cytokines also significantly raised in the experimental chickens (*p* < 0.05). The results shown that EmARM-β effectively promoted the expression of Th1 cytokines.

### The Cellular and Humoral Immune Response Induced by EmARM-β in Chickens

**Figure 6:**
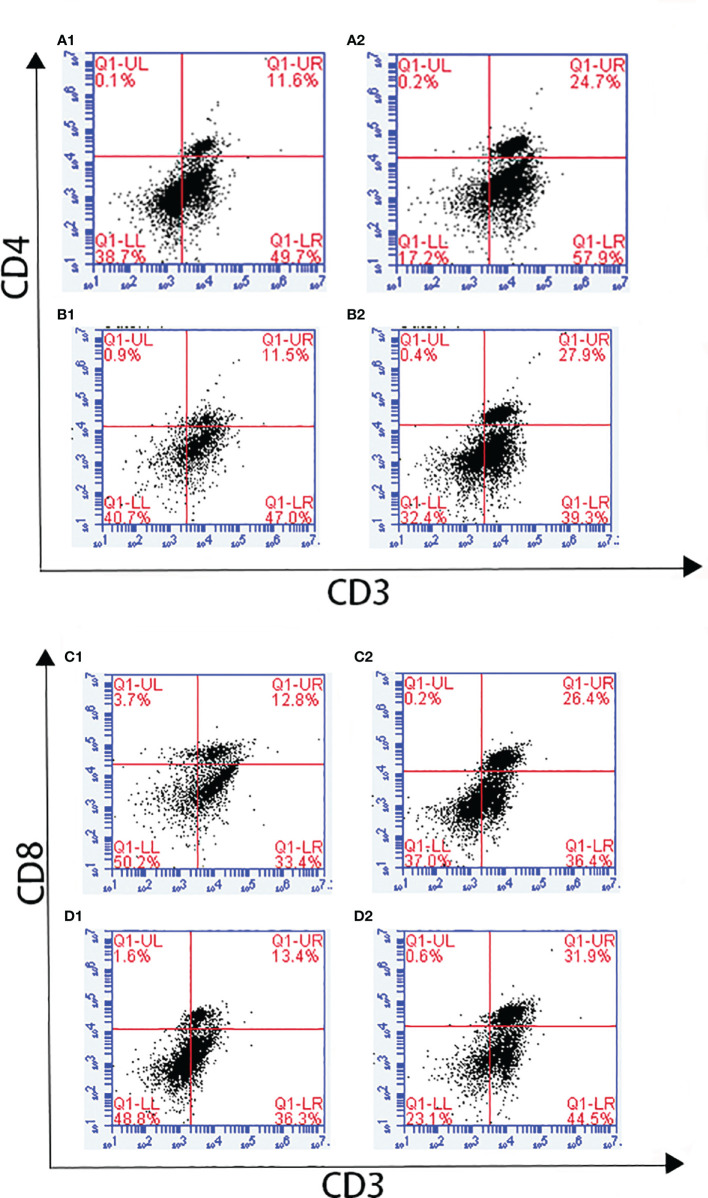
The proportion of T lymphocyte subset in spleen of pET-28a-EmARM-β immunized chickens. **(A)** Determination of CD4^+^/CD3^+^ T lymphocyte in immunized chicken spleen at 21 days old. **(B)** Determination of CD4^+^/CD3^+^ T lymphocyte in immunized chicken spleen at 28 days old. **(C)** Determination of CD8^+^/CD3^+^ T lymphocyte in immunized chicken spleen at 21 days old. **(D)** Determination of CD8^+^/CD3^+^ T lymphocyte in immunized chicken spleen at 28 days old. 1: PBS (negative control). 2: The ratio of T lymphocytes in spleens from chickens immunized with recombinant protein pET-28a-EmARM-β.

**Figure 7:**
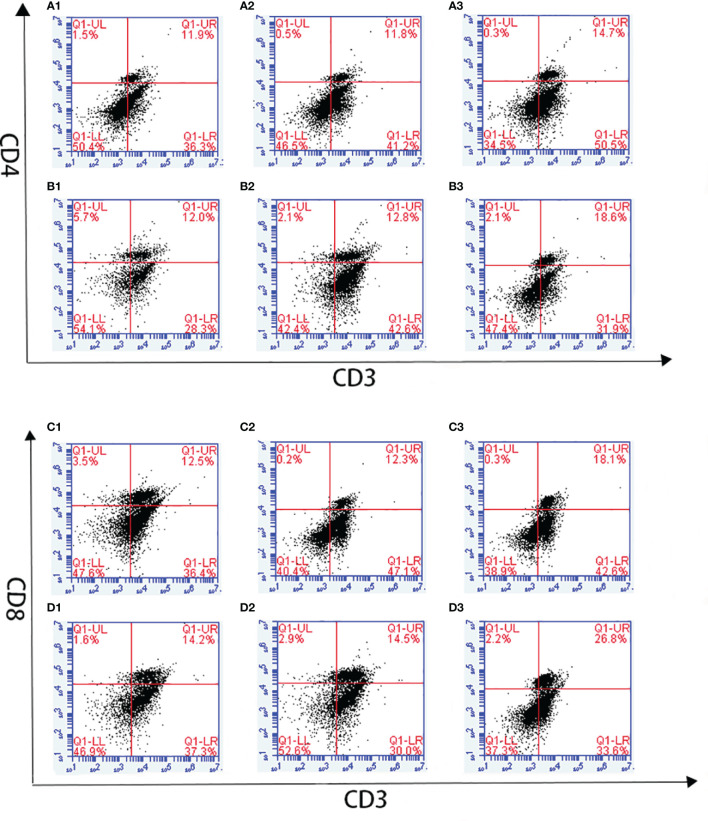
The proportion of T lymphocyte subsets in spleen of pVAX1-EmARM-β immunized chickens. **(A)** Determination of CD4^+^/CD3^+^ T lymphocyte in immunized chicken spleen at 21 days old. **(B)** Determination of CD4^+^/CD3^+^ T lymphocyte in immunized chicken spleen at 28 days old. **(C)** Determination of CD8^+^/CD3^+^ T lymphocyte in immunized chicken spleen at 21 days old. **(D)** Determination of CD8^+^/CD3^+^ T lymphocyte in immunized chicken spleen at 28 days old. 1: PBS (negative control). 2: The ratio of T lymphocytes in chicken spleen immunized with plasmid pVAX1. 3: The ratio of T lymphocytes in spleens from chickens immunized with recombinant plasmid pVAX1-EmARM-β.

**Figure 8:**
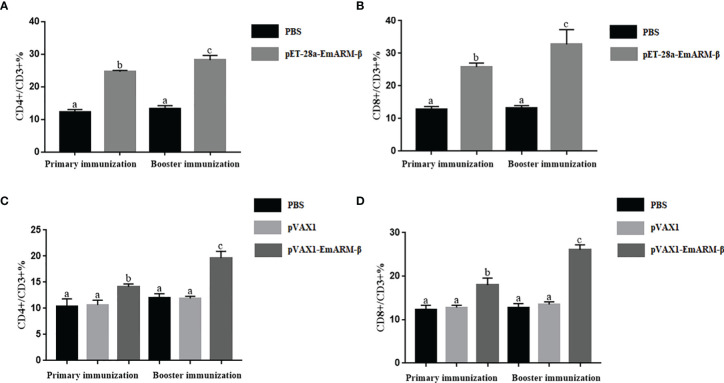
The ratio of T lymphocyte subset in EmARM-β immunized chicken spleens. **(A)** The ratio of CD4^+^/CD3^+^ T lymphocyte subset in spleens from chickens immunized with recombinant protein pET-28a-EmARM-β. **(B)** The ratio of CD8^+^/CD3^+^ T lymphocyte subset in spleens from chickens immunized with recombinant protein pET-28a-EmARM-β. **(C)** The ratio of CD4^+^/CD3^+^ T lymphocyte subset in spleens from chickens immunized with recombinant plasmid pVAX1-EmARM-β. **(D)** The ratio of CD8^+^/CD3^+^ T lymphocyte subset in spleens from chickens immunized with recombinant plasmid pVAX1-EmARM-β. The small letters (a-c) indicate difference between groups in the same panel. The same letter indicates no significant difference between different groups (p > 0.05). The different letter indicates significant difference between different groups (p < 0.05).

**Figure 9:**
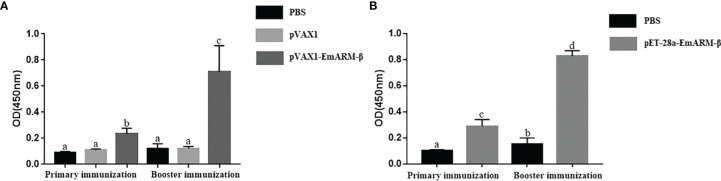
Specific IgG titers in chicken serum induced by EmARM-β. **(A)** Serum IgG titers induced by recombinant plasmid pVAX1-EmARM-β. **(B)** Serum IgG titers induced by recombinant protein pET-32a-EmARM-β. The small letters (a-c) indicate difference between groups in the same panel. The same letter indicates no significant difference between different groups (p > 0.05). The different letter indicates significant difference between different groups (p < 0.05).

Changes in T lymphocyte subpopulations and antibody response induced by EmARM-β antigen were detected by flow cytometry and indirect ELISA, respectively. The collection of spleens and heart blood was performed on the 21^st^ day and 28^th^ day to prepare splenocytes and chicken serum. Compared with control groups, a significant increase was observed in the proportions of CD4^+^/CD3^+^ and CD8^+^/CD3^+^ T cell subsets of experimental groups (*p* < 0.05) (**Figures 6**–**8**). The proportions of T cell subsets in experimental groups at the primary immunization increased higher than at the booster immunization. In comparison, the differences between control groups were not significant (*p >* 0.05). A similar change pattern was observed on antigen-specific IgG antibody levels (**Figure 9**). The treatment with recombinant protein or plasmid significantly promoted serum IgG levels when compared with control groups (*p* < 0.05). The antibody level at the booster immunization was much higher than that at the primary immunization. The results indicated EmARM-β antigen could significantly augment the production of CD4^+^ and CD8^+^ T lymphocyte and serum IgG antibodies.

### Protective Efficacy Assessment of EmARM-β Against *E. maxima* Infections

The protective efficacy of EmARM-β vaccines was assessed by determining enteric lesion score, oocysts output, weight gain, and ACI. As shown in **Table 2**, the weight gain of chickens from the unchallenged control group was significantly higher than challenged and pVAX1-treated controls (*p* < 0.05). There was no significant difference between challenged controls and pVAX1 control (*p >* 0.05). In contrast, experimental groups immunized with rEmARM-β or recombinant pVAX1-EmARM-β plasmid showed a significant restoration in weight loss compared with challenged control and pVAX1-treated control (*p* < 0.05).

**Table 2:** Protective efficacy assessment of EmARM-β against *E. maxima* infections.

Trial	Groups	Weight gain(g)	Mean lesion score	Mean OPG ×10^5^	Oocyst decrease rate %	ACI
1	Unchallenged control	42.13 ± 28.53^a^	0 ± 0^a^	0 ± 0^a^	100^a^	200
	Challenged control	18.90 ± 4.24^c^	2.41 ± 0.50^c^	1.90 ± 0.68^c^	0^c^	83.9
	rEmARM-β	36.38 ± 16.30^a^	1.58 ± 0.50^b^	0.45 ± 0.18^b^	76.31	171.91
2	Unchallenged control	46.24 ± 13.17^a^	0 ± 0^a^	0 ± 0^a^	100^a^	200
	Challenged control	18.60 ± 8.33^c^	2.44 ± 0.57^c^	2.05 ± 0.95^c^	0^c^	76.74
	pVAX1 control	21.84 ± 7.84^c^	2.37 ± 0.56^c^	1.94 ± 0.58^c^	5.36	85.83
	pVAX1-EmARM-β	38.79 ± 6.50^b^	1.55 ± 0.57^b^	0.42 ± 0.23^b^	79.51	168.65

Value = mean± S.D., n=20. Trial 1 and Trial 2 were conducted separately, the significance of data between these 2 trials were incomparable. The same letter (a-c) in the same columns indicates no significant difference between data in the same trial (p > 0.05). The different letter (a-c) in the same columns indicates significant difference between data in the same trial (p < 0.05).

The enteric lesion of the challenged chickens was also shown in **Table 2**. Lesion scores of two experimental groups showed a significant decrease compared with challenged control and pVAX1-treated control (*p* < 0.05). In addition, chickens immunized with pVAX1 plasmid scored similarly with blank birds (*p >* 0.05). A similar observation was also identified in the results of oocysts per gramme (OPG) value (**Table 2**). Compared with challenged and pVAX1 controls, experimental groups showed significantly lower mean oocysts count and higher oocyst decrease ratio (*p* < 0.05).

The anticoccidial index in **Table 2** also indicated protection efficacy induced by recombinant EmARM-β vaccines. Compared with challenged and pVAX1-treated controls, groups vaccinated with EmARM-β vaccines exhibited significantly higher ACI of 168.65 and 171.91, respectively. These results indicated that rEmARM-β and pVAX1-EmARM-β plasmid provided moderate protection against challenge infection with *E. maxima*.

## Discussion

Avian coccidiosis is commonly considered an economically damaging disease, seriously affecting the production efficiency of domestic chickens. Anticoccidial drugs and live vaccines immunization are currently employed as main control strategies. However, neither of them can go far due to their inherent drawbacks. Therefore, more effective and safer methods are imminently needed. The third-generation anticoccidial vaccines have recently aroused widespread public concern. Cheaper, safer, easier to massively produce, as well as more convenient to transportation and storage make novel vaccines more competitive than traditional ones (20, 21).

Unfortunately, the exploration of feasible candidate antigens is always a hard nut to crack in the development of vaccines. Expression library technology might be one of the novel strategies which could solve this problem. Among them, the cDNA expression library stands out for its unique advantages. Namely, this kind of library is composed of the genes expressed, thus making the selection of the stage-specific antigens possible (22). Zhu et al. screened 3 immunoprotective genes from the *Eimeria acervulina* cDNA expression library derived from sporozoite stage and evaluated the immunoprotective efficacy of two genes cSZ-JN1 and cSZ-JN2. (23, 24). Jenkins and colleagues constructed an *E. acervulina* cDNA library utilizing sporozoites and merozoites and identified a surface antigen cMZ-8, which can effectively activate T lymphocytes *in vitro* (25). Réfega et al. also found three immunodominant genes from the built *Eimeria tenella* cDNA library of sporozoites and the f irst generation merozoite stages (26). All these results indicated the practicality and potential of cDNA expression library technology. In our preliminary work, we constructed a cDNA expression library of *E. maxima* sporozoites (27). Through five rounds of cDNA library immunization, alongside detecting specific transcription factors of Th1 and Th2 cells and related cytokines after each immunization, we obtained a stimulating antigen of Th1 cytokines (EmARM-β) (unpublished results). In the present study, the protective efficacy of this antigen was assessed. The results indicated that it could induce moderate protection against *E. maxima* infection and possess the potential to become a candidate antigen for novel vaccine development.

Cell-mediated immunity was universally known to play a dominant role against coccidiosis (28, 29). Th cells are no doubt a vital subpopulation among all immune cells. They are activated by mature dendritic cells to secrete various cytokines with specific functions. They are then involved in the initiation and regulation of host immune response and assist other immunocytes in resisting invading pathogens (30, 31). Th1, Th2, iTreg, and Th17 cells, these four major Th cell subsets play important roles in host cellular immunity (6). Typically produced by Th1 cells, cytokines such as IFN-γ and IL-2 can effectively protect the host from intracellular pathogens. Therefore, inhibition of Th1 cytokines will provide an opportunity for pathogen invasion. A spectrum of findings confirmed this phenomenon during infections of certain parasites (32, 33). For example, in *Trypanosoma cruzi* invasion, *the* MAPK pathway was activated by some specifically-secretory regulatory molecules, which resulted in the functional inhibition of Th1 cells (34). *Leishmania* can directly activate the MAPK signal pathway and inhibit the expression of IL-12 (Th1 positive regulation), thus inhibiting Th1 cell functions (35). Besides, Th1 cell inhibition also exists in chickens infected by *Eimeria* species. Cornelissen et al. revealed that infection by coccidiosis led to a down-regulation of Th1 cytokines (36). On the contrary, an antigen that can stimulate Th1 cytokines may be a promising candidate for vaccine development. In the present study, we discovered the significant stimulating function of the EmARM-β antigen to Th1 cytokines (IL-2 and IFN-γ) in chickens. Meanwhile, as a hallmark cytokine expressed by Th2 cells, IL-4 was also significantly stimulated by rEmARM-β and pVAX1-EmARM-β plasmid *in vivo*. This result indicated that the EmARM-β could induce robust Th1 and Th2 immune responses, which are super important in resisting coccidiosis.

T-bet and GATA3 are vital transcription factors for the differentiation of Th1 and Th2 cells, respectively (37, 38). T-bet is highly expressed in the early phase of Th1 cell development but silenced during Th2 cell development (39). It can trigger the differentiation of CD4^+^ T cell to Th1 type through activating the IFN-γ gene, suppressing the differentiation to Th2 cell (40–42). On the contrary, the expression of GATA3 increases in the differentiation of CD4^+^ T cell to Th2 type (37). In this study, we detected T-bet and GATA3 mRNA levels in the PBMC of chickens. We found rEmARM-β protein and recombinant plasmid exhibited a stimulating effect on both transcription factors *in vivo.* The specific reason needs further investigation.

Although Th1 response plays a predominant role in the immune response against avian coccidiosis, other avian T cell lineages such as Th17 and T regulatory cells are involved in maintaining gut homeostasis during the infection of avian coccidia (58). As a proinflammatory immunocyte, Th17 is mainly responsible for inducing inflammation in the infection site during the acute stage of coccidiosis (43, 44). An excessive inflammatory response will undoubtedly damage the intestinal tract of infected chickens, while Treg cells can alleviate intestinal injury by suppressing the production of Th17 cells (6). Apart from Th17 cells, Treg cells can suppress varied activated immune cells, but excessive enhancement of this suppressed activity will create an excellent opportunity for pathogens invasion. Hence one can see that balance between Treg and proinflammatory cytokines is indispensable for appropriate inflammatory responses (6, 45). In this study, we can observe significantly increased levels of IL-10 and TGF-β *in vivo*. This can be attributed to the enhanced activity of Treg cells to repair injured intestines.

The function of cellular immunity depends largely on the coincident work of T cells, macrophages, natural killer (NK) cells, and various cytokines. Among them, responses induced by T cells act dominantly in cell-mediated protection against coccidiosis due to the intracellular parasitism characteristics of coccidia (46, 47). Two members of the T cell family, CD8^+^ and CD4^+^ T lymphocytes, contribute differently to the protection against infections by *Eimeria* species. The former mediates immune protection dependent mainly on killing and eliminating the infected cells or secreting cytokines, while the latter is responsible for activating NK cells and macrophages and producing Th cells (48, 49). An increase in the level of CD4^+^ and CD8^+^ T lymphocytes was observed in chickens immunized with *E. tenella* 3-1E protein, which uncovered the involvement of these two T cell subpopulations in resisting coccidiosis (50). Similarly, compared with controls, the proportion of T lymphocyte subsets increased significantly in EmARM-β-immunized chickens in this study. It indicated a stimulating function of EmARM-β to cell-mediated response as well as T cell proliferation and differentiation.

Humoral immunity has been considered a minor function against infections by *Eimeria* species for a long time (51, 52). However, many researchers agree on the relevance between antibody immune response and the protection against coccidiosis (53, 54). A convincing example lies in the protection provided by CoxAbic^®^ to the offspring from parent chickens (55). In addition, in ovo immunization with *E. acervulina* 3-1E protein supplemented by cytokine genes can induce high antibody titers in chickens (56). In this study, an significant increase of the level of specific antibodies in immunized chickens was observed compared with that of control groups, indicating that antibody responses induced by EmARM-β vaccines might be involved in coccidiosis resistance.

Protective efficacy of EmARM-β was evaluated through immunization-challenge trials in the forms of recombinant protein (rEmARM-β) and recombinant plasmid (pVAX1-EmARM-β). Immunization with EmARM-β could alleviate enteric lesion and body-weight loss, reduce oocysts output of the chickens infected by *Eimeria maxima*. The ACIs of the immunized groups were beyond 160, showing moderate protection against *Eimeria maxima*. This result indicates that EmARM-β is a promising candidate antigen for anticoccidial vaccine development. However, some strategies researches could be adopted to improve its protective efficacy. For example, protective efficacy of EmARM-β could be improved by the addition of cytokines as adjuvant (10, 57). The non-injection administration routes also remain to be further investigated to make it more practical in poultry farm.

In summary, EmARM-β was verified to promote the expression of Th1 cytokines significantly, thereby inducing both Th1 and Th2 immune responses. Besides, it could provide moderate protection against challenge infection of *E. maxima* in the forms of recombinant protein and recombinant plasmid. These results indicated that EmARM-β is hopeful to be a candidate antigen for the development of anti-coccidiosis vaccine.

## Data Availability Statement

The datasets presented in this study can be found in online repositories. The names of the repository/repositories and accession number(s) can be found below: https://www.ncbi.nlm.nih.gov/, XM_013482460.1.

## Ethics Statement

The animal study was reviewed and approved by The Committee on Experimental Animal Welfare and Ethics of Nanjing Agricultural University.

## Author Contributions

Conceptualization: XS and XL; Investigation: JL, YZ, and CC; Methodology: JL; Resources: LX, XL, XS, and RY; Software: YZ and CC; Supervision: XS; Validation: CC and LX; Visualization: MW; Writing – original draft: CC; Writing – review and editing: XS and ML. All authors contributed to the article and approved the submitted version.

## Funding

This work was supported by the National Natural Science Foundation of China (Grant No. 31972705 and 31672545).

## Conflict of Interest

The authors declare that the research was conducted in the absence of any commercial or financial relationships that could be construed as a potential conflict of interest.

## Publisher’s Note

All claims expressed in this article are solely those of the authors and do not necessarily represent those of their affiliated organizations, or those of the publisher, the editors and the reviewers. Any product that may be evaluated in this article, or claim that may be made by its manufacturer, is not guaranteed or endorsed by the publisher.
